# Effect of Standardized Nutritional Intervention in Patients with Nasopharyngeal Carcinoma Receiving Radiotherapy Complicated with Diabetes Mellitus

**DOI:** 10.1155/2022/6704347

**Published:** 2022-06-15

**Authors:** Yuhong Ge

**Affiliations:** Department of Head and Neck Comprehensive Radiation Therapy, Affiliated Tumor Hospital of Xinjiang Medical University, Urumqi, China

## Abstract

**Objective:**

To evaluate the effect of standardized nutritional intervention in patients with nasopharyngeal carcinoma receiving radiotherapy complicated with diabetes mellitus and the impact on quality of life.

**Methods:**

From January 2019 to December 2020, 100 diabetic patients with nasopharyngeal carcinoma receiving radiotherapy were assessed for eligibility and recruited. They were concurrently and randomly assigned (1 : 1) to receive either conventional nursing (control group) or standardized nutritional intervention (observation group). The outcomes include clinical efficacy and quality of life.

**Results:**

Standardized nutritional intervention was associated with significantly lower levels of fasting blood glucose (FBG), 2 h postprandial blood glucose (2hPBG), and glycated hemoglobin (HbA1c) versus conventional nursing (*P* < 0.001). The patients given standardized nutritional intervention showed significantly higher hemoglobin (Hb), prealbumin (PA), and albumin (ALB) levels versus those given conventional nursing at 4 weeks after the start of radiotherapy and at the end of radiotherapy (*P* < 0.001). The two groups showed similar Morisky scores before intervention (*P* > 0.05). After intervention, the observation group outperformed the control group in terms of treatment compliance (*P* < 0.05). Standardized nutritional intervention provided patients with a significantly better quality of life versus conventional nursing (*P* < 0.05). Standardized nutritional intervention was associated with a significantly lower incidence of adverse events and higher nursing satisfaction versus conventional nursing (*P* < 0.05).

**Conclusion:**

Standardized nutritional intervention for patients with nasopharyngeal carcinoma given radiotherapy complicated with diabetes mellitus can efficiently restore the normal nutritional status of patients, reduce the complications of radiotherapy, and improve the quality of life of patients, so it is worthy of wide clinical application.

## 1. Introduction

Nasopharyngeal carcinoma is a highly prevalent malignant tumor, with a predominant incidence of head and neck malignant tumors and a high mortality rate [1]. It is a common malignant tumor in the south of China, with the age of onset mostly between 30 and 60 years [2]. The prevalence of nasopharyngeal cancer in China is about 39.84 per ten thousand, with an incidence of about 14.68 per ten thousand and a mortality of about 8.73 per ten thousand [3]. Its clinical symptoms mostly include nasal congestion, bloody nasal discharge, congestion in the ear, hearing loss, diplopia, and headache [4]. Given its moderate sensitivity to radiation therapy, radiotherapy is the current treatment of choice for nasopharyngeal carcinoma. Diabetes mellitus is a group of metabolic diseases characterized by hyperglycemia, and the long-term presence of hyperglycemia leads to chronic damage and dysfunction of various organs and tissues, such as the eyes, kidneys, heart, blood vessels, and nerves [4]. With the continuous improvement of the living standard, the dietary structure and lifestyle of people have undergone significant changes, resulting in a marked increase in the prevalence of diabetes [5]. An epidemiological survey conducted by Mainous et al. found the prevalence of uropathy in American adults aged 40-49, 50-59, and 60-69 years to be 4.5%, 11.4%, and 15.1%, respectively [6], and it has been shown that 9% of cancer patients had diabetes mellitus at the time of cancer diagnosis, suggesting a close association between long-term diabetes mellitus and tumorigenesis [7, 8]. With the continuous application of new technologies such as intensity-modulated radiation therapy (IMRT) in clinical practice, the survival of nasopharyngeal cancer has markedly improved [9]. However, radiotherapy may cause collateral damage to normal tissue cells, and complications of radiotherapy including radioactive oral mucositis, radioactive esophagitis, radioactive skin damage, and restricted mouth opening seriously compromise the patients' nutritional status and quality of life. Standardized nutritional intervention is a new management approach that provides patients with nutrients and energy to perform the metabolic and immunomodulatory functions of nutrients [10, 11]. Moreover, it helps the patients master reasonable dietary habits and lifestyles and prevent related complications, thereby enhancing their quality of life. This study was conducted to develop nutritional plans for diabetic patients undergoing radiotherapy for nasopharyngeal carcinoma through standardized nutritional intervention. The results are as follows.

## 2. Materials and Methods

### 2.1. Baseline Data

From January 2019 to December 2020, 100 diabetic patients with nasopharyngeal carcinoma receiving radiotherapy were assessed for eligibility and recruited. They were concurrently and randomly assigned to control group (*n* = 50) or observation group (*n* = 50). The baseline characteristics of the observation group (31 males, 19 females, aged 35-71 years, mean age of [45.02 ± 3.68] years, course of disease of [3-11] years, and mean course of disease of [7.02 ± 3.64] years) were comparable with those of the control group (35 males, 15 females, aged 36 - 70 years, mean age of [45.45 ± 3.34] years, course of disease of [3-11] years, and mean course of disease of [7.56 ± 3.17] years) (*P* > 0.05) (Table 1). The studies involving human participants were reviewed and approved by Affiliated Tumor Hospital of Xinjiang Medical University. The patients provided their written informed consent to participate in this study.

### 2.2. Inclusion and Exclusion Criteria

Inclusion criteria: ① patients with pathologically confirmed primary nasopharyngeal carcinoma; ② with diabetes mellitus diagnosed before radiotherapy as per the 1999 WHO diagnostic criteria for diabetes mellitus; and ③ with normal consciousness to conduct normal communication.

Exclusion criteria: ① patients with other primary tumors; ② with withdrawal of consent; and ③ with severe psychiatric disorders.

### 2.3. Nursing Methods

Patients in the control group were given conventional nursing, including therapeutic care, disease health education, dietary guidance, and medication care.

Patients in the observation group received standardized nutritional intervention. A nutrition management team consisting of nutrition specialist nurses, doctors, and dietitians was established. The patients were regularly educated about nutrition knowledge of nasopharyngeal carcinoma and diabetes, the detriments of the disease, the necessity of blood glucose control, and the importance of individualized nutritional interventions. Nutritional dietary guidance was provided through online channels to address radiotherapy nutrition-related issues [12] and collect follow-up data. The patients were encouraged to perform patient self-management, and the nutrition nurses instructed the patients to accurately record their weight and the amount and type of food for their daily consumption. The intervention was continued for one month. And they all were followed-up for six months.

### 2.4. Evaluation Criteria

① Glucose levels: the blood glucose values after dietary care were compared between the two groups, and the patients' fasting blood glucose (FBG), 2 h postprandial blood glucose (2hPBG), and glycated hemoglobin (HbA1c) levels after the intervention were determined for analyses. 5 ml of fasting venous blood in the morning before and after the intervention of the two groups of women was collected, centrifuged (Avanti JXN-30/26, Beckman Company) at 3000 r/min for 10 min, and then, an automatic biochemical analyzer (Nanjing Baden Medical Co., Ltd.) was used to measure maternal FBG and 2 h postprandial blood glucose (2 h PBG). High-performance liquid chromatography (HPLC) was used to detect the HbA1c levels of the two groups before and after the intervention, and the normal value was 4-6%.

② Nutritional status: serum nutritional indices, including hemoglobin (Hb), prealbumin (PA), and albumin (ALB) levels, were determined and compared between the two groups of patients before the intervention, at 4 weeks after the start of radiotherapy, and at the end of radiotherapy.

③ Compliance: the Morisky compliance scale [13, 14] was used to evaluate the patients' compliance with treatment before and after the nursing intervention in four aspects: medication compliance, body mass control, diet control, and appropriate exercise, with a total score of 50 points. The higher the score, the better the compliance.

④ Quality of life: the quality of life of patients in both groups before and after radiotherapy was evaluated by the FACT-H&N scale [15]. The FACT-H&N consists of 11 head and neck entries (HN1 to 11). GP1 to GP7, GE1 to GE6, HN2, HN3, HN6, HN8, and HN9 are reverse entries, and the rest are positive entries. Scores for positive entries = (0 + answer option number); score for reverse entries = (4 − answer option number). Higher scores represent better life quality.

⑤ Adverse events: complications such as radioactive skin damage, radioactive mucositis, oral ulcers, difficulty in opening mouth, otitis media, and dry mouth were monitored and recorded in both groups.

⑥ Satisfaction: the nursing satisfaction questionnaire (including the attitude of medical staff, the efficiency of medical staff, and explanation of diseases by medical staff) developed by our hospital was used, containing a total of four items (highly satisfied, satisfied, less satisfied, and dissatisfied).

### 2.5. Statistical Analysis

The SPSS22.0 software was used for data analyses, and GraphPad Prism 8 was used for image rendering. The count data were expressed as [*n* (%)] and processed using the chi-square test. The measurement data were expressed as ($\bar{x}\pm s$) and analyzed using the *t*-test. Differences were considered statistically significant at *P* < 0.05.

## 3. Results

### 3.1. Blood Glucose Levels

Standardized nutritional intervention was associated with significantly lower levels of FBG, 2hPBG, and HbA1c (6.58 ± 0.64, 8.95 ± 0.35, 8.06 ± 2.35) versus conventional nursing (7.01 ± 0.62, 9.86 ± 0.65, 11.98 ± 2.36) (*P* < 0.001) (Table 2).

### 3.2. Nutritional Status

Before radiotherapy, the Hb, PA, and ALB levels of the two groups were similar (*P* > 0.05). The patients given standardized nutritional intervention showed significantly higher Hb, PA, and ALB levels (131.17 ± 10.77, 268.07 ± 22.32, 42.78 ± 4.12/130.55 ± 10.86, 267.25 ± 19.78, 41.17 ± 3.76) versus those given conventional nursing at 4 weeks after the start of radiotherapy and at the end of radiotherapy (*P* < 0.001) (Table 3).

### 3.3. Compliance

**Figure 1:**
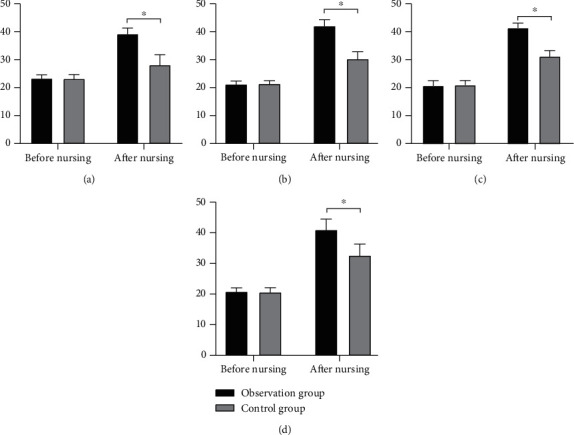
Comparison of Morisky scores. Note: ^∗^ indicates the differences between the two groups were statistically significant; (a) is the comparison of control body mass; (b) is the comparison of medication compliance; (c) is the comparison of appropriate exercise; (d) is the comparison of control diet.

The two groups showed similar Morisky scores before intervention (*P* > 0.05). After intervention, the observation group (39.21 ± 2.13, 41.67 ± 2.25, 41.99 ± 1.84, 43.07 ± 3.68) outperformed the control group (28.15 ± 3.68, 30.01 ± 2.54, 31.65 ± 2.17, and 34.26 ± 3.98) in terms of control body mass, medication compliance, appropriate exercise, and diet control (*P* < 0.05) (Figure 1).

### 3.4. Quality of Life

Standardized nutritional intervention (91.27 ± 7.68) provided patients with a significantly better quality of life versus conventional nursing (79.88 ± 5.37) (*P* < 0.05) (Table 4).

### 3.5. Adverse Events

Standardized nutritional intervention (2 [4.00%] cases of radioactive skin injury, 2 cases [4.00%] of radioactive mucositis, 3 [6.00%] cases of oral ulcers, 1 case [2.00%] of difficulty in opening the mouth, and 2 cases [4.00%] of dry mouth) was associated with a significantly lower incidence of adverse events versus the conventional nursing (8 cases [16.00%] of radioactive skin injury, 9 [18.00%] cases of radioactive mucositis, 12 [24.00%] cases of oral ulcer, 7 [14.00%] cases of difficulty in opening mouth, 4 [8.00%] cases of otitis media, and 9 [18.00%] cases of dry mouth) (*P* < 0.05) (Table 5).

### 3.6. Nursing Satisfaction

**Figure 2:**
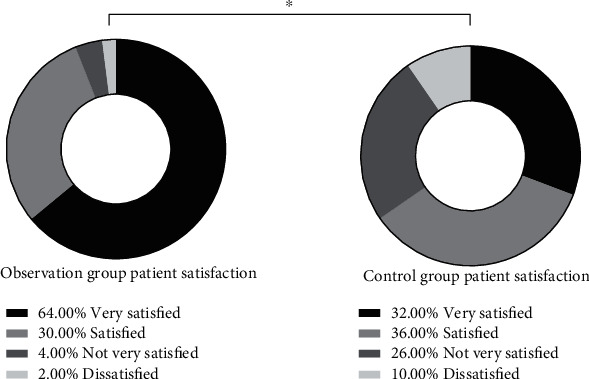
Comparison of SF-36 scores. Note: ^∗^ indicates the differences between the two groups were statistically significant.

Standardized nutritional intervention (94.00%, including 32 [64.00%] cases of highly satisfied, 15 [30.00%] cases of satisfied, 2 [4.00%] cases of less satisfied, and 1 [2.00%] case of dissatisfied) resulted in a significantly higher nursing satisfaction versus conventional nursing (64.00%, including 16 [32.00%] cases of highly satisfied, 18 [36.00%] cases of satisfied, 13 [26.00%] cases of less satisfied, and 5 [10.00%] cases of dissatisfied) (*P* < 0.05) (Figure 2).

## 4. Discussion

In recent years, the prevalence of diabetes mellitus has increased year by year as the living standard of the population improves with the development of the economy. Epidemiological surveys have revealed that 9% of cancer patients have diabetes mellitus at the time of cancer diagnosis, suggesting a strong association between long-term diabetes mellitus and carcinoma [8, 16]. Nasopharyngeal carcinoma is a common malignancy in clinical practice with a high lethality rate [17]. The optimal clinical treatment modality is radiotherapy [18]. However, radiotherapy may result in serious complications such as radiation oral mucositis, pain, and restricted mouth opening that impair feeding and result in insufficient nutritional intake, water, and electrolyte imbalance [19, 20]. Normative nutritional intervention is a new management model that supplies nutrients and energy to patients.

The results in the present study showed significantly lower levels of FBG, 2hPBG, and HbA1c in patients receiving standardized nutritional intervention versus conventional nursing, suggesting that personalized dietary care of patients through standardized nutritional intervention effectively ameliorates the patients' blood glucose levels and improves the efficiency of disease control [21]. All these might be attributed to the fact that the diet formulated by standardized nutritional intervention is scientific and reliable, which contributes greatly to the control of blood sugar especially targeting patients undergoing radiotherapy for nasopharyngeal carcinoma. Also, the patients were regularly educated about nutrition knowledge of nasopharyngeal carcinoma and diabetes, the detriments of the disease, further reinforcing their consciousness towards nasopharyngeal carcinoma, and diabetes-related nutrition. Previous research has indicated a better nutritional status of patients receiving standardized nutritional intervention versus conventional nursing [22], which was consistent with the results of the present study. Moreover, standardized nutritional intervention herein was associated with higher Morisky scores and quality of life and a lower incidence of adverse events versus conventional nursing. Standardized nutritional intervention for patients undergoing synchronous radiotherapy for nasopharyngeal carcinoma can effectively improve patient compliance with promising diet control, blood glucose control, and exercise management. Possibly, the standardized nutritional intervention encouraged to perform patient self-management, and patients thus build a habit of monitoring and managing themselves, which serves as a contributor to prominent outcomes.

Here, the observation group showed significantly higher nursing satisfaction versus the control group. Radiotherapy for nasopharyngeal carcinoma complicated with diabetes mellitus features a long course and various adverse events, which seriously compromises the physical and mental health and quality of life of patients. The standardized nutritional intervention provides patients with regular nutritional education, regular follow-up, and the design of reasonable diet plans according to patients' specific conditions, which boosts treatment quality versus conventional nursing interventions. Consistently, the findings of the present study were similar to the previous ones [12, 21].

In conclusion, standardized nutritional intervention for patients with nasopharyngeal carcinoma combined with diabetes mellitus during radiotherapy can efficiently maintain the normal nutritional status of patients, reduce the complications of radiotherapy, and improve the quality of life of patients, so it is worthy of wide clinical application.

## Figures and Tables

**Table 1:** Comparison of baseline data ($\bar{x}\pm s$).

Groups	*n*	Male	Female	Age	Mean age	Course of disease	Mean course of disease
Observation group	50	31	19	35-71	45.02 ± 3.68	3-11	7.02 ± 3.64
Control group	50	35	15	36-70	45.45 ± 3.34	3-11	7.56 ± 3.17
*t*	—	—	—	—	0.612	—	0.791
*P*	—	—	—	—	0.542	—	0.431

**Table 2:** Comparison of blood glucose levels after intervention ($\bar{x}\pm s$).

*n*	*n*	FBG (mmol/L)	2Hpbg (mmol/L)	HbA1c (%)
Observation group	50	6.58 ± 0.64	8.95 ± 0.35	8.06 ± 2.35
Control group	50	7.01 ± 0.62	9.86 ± 0.65	11.98 ± 2.36
*t*	—	3.412	8.716	8.323
*P*	—	0.001	<0.001	<0.001

Note: FBG: fasting blood glucose; 2hPBG: two-hour postprandial blood glucose; HbA1c: glycated hemoglobin.

**Table 3:** Comparison of nutritional status related blood indices ($\bar{x}\pm s$).

Groups	*n*	Timepoint	Hb (g/L)	PA (mg/L)	ALB (g/L)
Observation group	50	Before intervention	132.65 ± 11.16	271.17 ± 23.43	44.02 ± 3.98
		At 4 weeks of intervention	131.17 ± 10.77	268.07 ± 22.32	42.78 ± 4.12
		At the end of intervention	130.55 ± 10.86	267.25 ± 19.78	41.17 ± 3.76
Control group	50	Before intervention	131.84 ± 12.04	270.61 ± 21.19	43.75 ± 4.23
		At 4 weeks of intervention	123.76 ± 11.25	234.95 ± 16.44	37.83 ± 4.33
		At the end of intervention	106.73 ± 9.88	210.07 ± 14.56	34.82 ± 1.77
*t*	—	Before intervention	0.349	0.125	0.329
		At 4 weeks of intervention	3.364	8.448	5.856
		At the end of intervention	11.472	16.462	10.805
*P*	—	Before intervention	0.728	0.901	0.743
		At 4 weeks of intervention	0.001	<0.001	<0.001
		At the end of intervention	<0.001	<0.001	<0.001

Note: *t*-values and *P* values are for the same period comparison between the two groups. Hb: hemoglobin; PA: prealbumin; ALB: albumin.

**Table 4:** Comparison of FACT-H&N scores ($\bar{x}\pm s$).

Groups	*n*	Before intervention	After intervention
Observation group	50	105.87 ± 5.65	91.27 ± 7.68^∗^
Control group	50	106.09 ± 5.73	79.88 ± 5.37^∗^
*t*	—	0.193	8.594
*P*	—	0.847	<0.001

Note: ^∗^ is a statistically significant difference between pre- and postintervention in the same group, *P* < 0.05.

**Table 5:** Comparison of incidence of adverse events (%).

Groups	*n*	Radioactive skin injury	Radioactive mucositis	Oral ulcers	Difficulty in opening mouth	Otitis media	Dry mouth
Observation group	50	2 (4.00)	2 (4.00)	3 (6.00)	1(2.00)	0 (0.00)	2 (4.00)
Control group	50	8 (16.00)	9 (18.00)	12 (24.00)	7 (14.00)	4 (8.00)	9 (18.00)
*t*	—	4.000	5.005	6.353	4.891	4.167	5.005
*P*	—	0.046	0.025	0.012	0.027	0.041	0.025

## Data Availability

The datasets used during the present study are available from the corresponding author upon reasonable request.
